# A combined predicting model for benign esophageal stenosis after simultaneous integrated boost in esophageal squamous cell carcinoma patients (GASTO1072)

**DOI:** 10.3389/fonc.2022.1026305

**Published:** 2022-12-22

**Authors:** Weitong Liu, Chengbing Zeng, Siyan Wang, Yizhou Zhan, Ruihong Huang, Ting Luo, Guobo Peng, Yanxuan Wu, Zihan Qiu, Derui Li, Fangcai Wu, Chuangzhen Chen

**Affiliations:** ^1^ Department of Radiation Oncology, Cancer Hospital of Shantou University Medical College, Shantou, China; ^2^ Department of Radiation Oncology, Jieyang People’s Hospital, Jeiyang, China; ^3^ Department of Radiation Oncology, Shenshan Central Hospital, Sun Yat-Sen Memorial Hospital, Sun Yat-Sen University, Shanwei, China; ^4^ Department of Otolaryngology-Head and Neck Surgery, The First Affiliated Hospital of Shantou University Medical College, Shantou, China

**Keywords:** esophageal cancer, esophageal stenosis, radiomics, chemoradiotherapy, radiotherapy

## Abstract

**Purpose:**

We aimed to develop a combined predicting model for benign esophageal stenosis (BES) after simultaneous integrated boost (SIB) with concurrent chemotherapy in patients with esophageal squamous cell carcinoma (ESCC).

**Methods:**

This study included 65 patients with EC who underwent SIB with chemotherapy. Esophageal stenosis was evaluated using esophagograms and the severity of eating disorders. Risk factors were investigated using univariate and multivariate analyses. Radiomics features were extracted based on contrast-enhanced CT (CE-CT) before treatment. The least absolute shrinkage and selection operator (LASSO) regression analysis was used for feature selection and radiomics signature construction. The model’s performance was evaluated using Harrell’s concordance index and receiver operating characteristic curves.

**Results:**

The patients were stratified into low- and high-risk groups according to BES after SIB. The area under the curves of the clinical model, Rad-score, and the combined model were 0.751, 0.820 and 0.864, respectively. In the validation cohort, the AUCs of these three models were 0.854, 0.883 and 0.917, respectively. The Hosmer-Lemeshow test showed that there was no deviation from model fitting for the training cohort (p=0.451) and validation cohort (p=0.481). The C-indexes of the nomogram were 0.864 and 0.958 for the training and validation cohort, respectively. The model combined with Rad-score and clinical factors achieved favorable prediction ability.

**Conclusion:**

Definitive chemoradiotherapy could alleviate tumor-inducing esophageal stenosis but result in benign stenosis. We constructed and tested a combined predicting model for benign esophageal stenosis after SIB. The nomogram incorporating both radiomics signature and clinical prognostic factors showed favorable predictive accuracy for BES in ESCC patients who received SIB with chemotherapy.

**Trial registration number and date of registration:**

Registered in www.Clinicaltrial.gov, ID: NCT01670409, August 12, 2012

## Introduction

Esophageal cancer (EC) is a common gastrointestinal malignancy, with squamous cell carcinoma (ESCC) being the predominant type. It has a high incidence in Eastern and Central Asia (1, 2). Patients with locally advanced disease, particularly those with unresectable tumors have an unsatisfactory prognosis, on account of a less than 30% 5-year survival rate (3). For locally advanced EC patients who reject or cannot tolerate surgery, concurrent chemoradiotherapy has been a standard recommendation due to higher long-term survival rates and insignificant differences in late toxicity compared to single radiotherapy (4).

Recently, a clinical approach known as simultaneous integrated boost (SIB) that delivers a higher dose fractionation to the gross tumor volume while delivering a lower dose fractionation to the clinical target volume has been approved as feasible with acceptable toxicities (5–7). We also explored this therapeutic mode for esophageal squamous cell carcinoma (ESCC) in a phase II clinical trial. Preliminary results demonstrated that tumor control and overall survival improved when compared with historical data (8). The long-term outcome has been reported in European Society for Therapeutic Radiology and Oncology (ESTRO), and the phase III clinical trial was currently being conducted. Although the tolerability of such treatment regimens was acceptable in these studies, their treatment-related late toxicities were not well established, particularly in the case of benign esophageal stenosis (BES). BES arises from various etiologies including peptic, radiation, and caustic injury. It differs from malignant esophageal stenosis due to tumor mass and can impair the patient’s quality of life and lead to serious complications like weight loss, malnutrition, and aspiration (9). The late toxicities of esophageal radiotherapy were predominantly manifested as benign stenosis and esophageal dysmotility (10, 11). Previous studies have shown that esophageal stenosis after conventional fractional radiotherapy of EC was correlated with the extent of the circumference involved (ECI), T stage, the longitudinal length of the tumor (LLT), and the wall thickness of the affected esophagus (12–14). Whether these factors continue to be related to BES after SIB has not been verified.

Radiomics, which extracted high-dimensional quantitative features from radiographic images to provides additional information on the heterogeneity and phenotype of tumor aggressiveness (15–17). It can be used for disease detection, cancer diagnosis, and treatment outcome prediction (18–23). Previous radiomics studies on EC have mainly focused on predicting tumor differentiation, staging, lymph node metastasis, and survival outcomes (24–27). To our knowledge, there has been no radiomics-based studies on toxicity prediction for high-dose radiotherapy in patients with ESCC.

Hence, this study sought to identify both clinical and radiomics features correlated with BES after SIB in patients with ESCC and develop a nomogram for prediction.

## Methods and materials

### Patients

**Figure 1:**
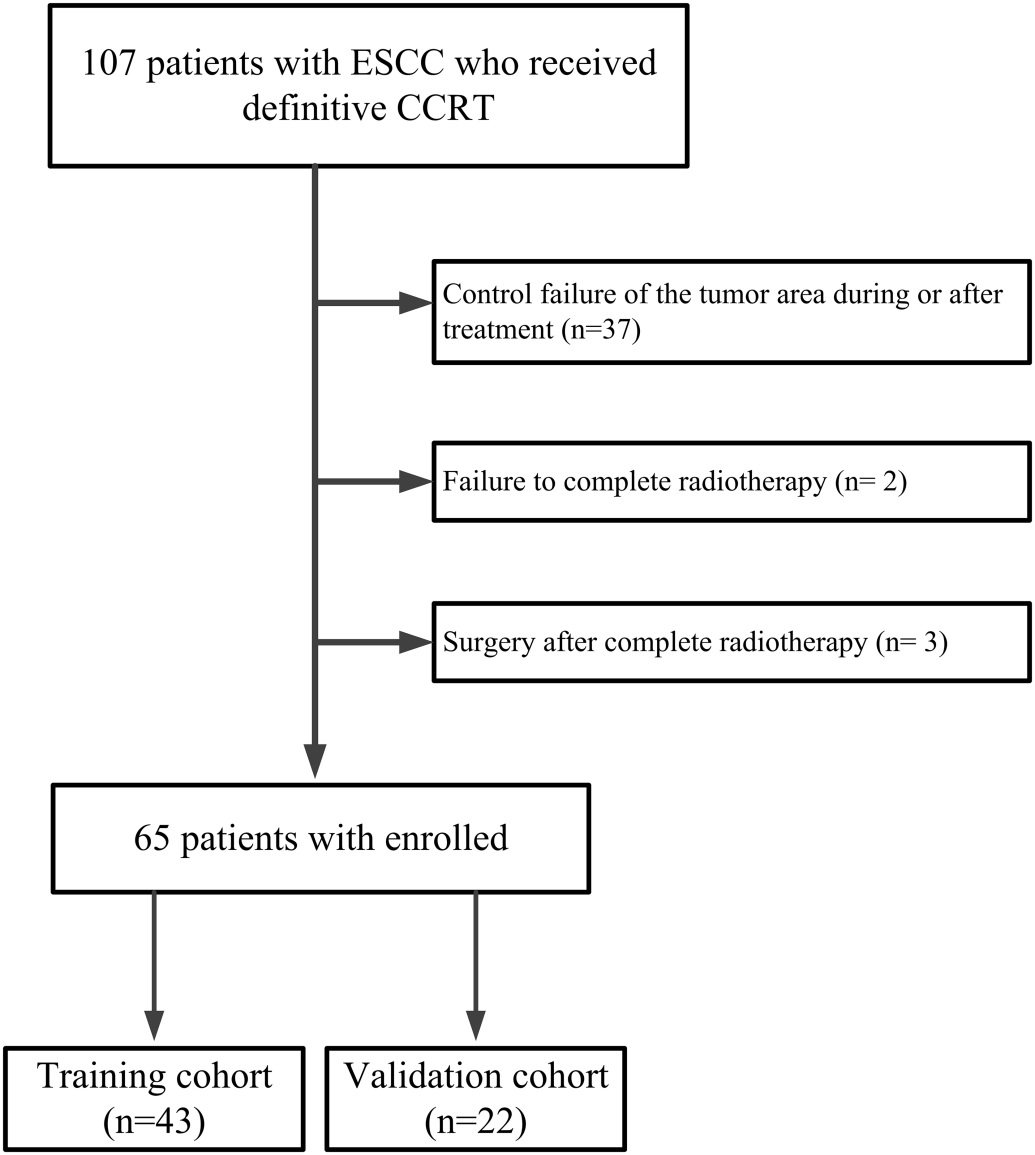
The workflow of Inclusion and Exclusion.

From August 2012 to January 2018, we investigated 107 patients with ESCC who received SIB with concurrent chemotherapy from a single-arm, prospective phase II clinical trial called “simultaneous modulated accelerated radiotherapy combined with chemotherapy for esophageal cancer” (clinical trial: NCT01670409) at the Cancer Hospital of Shantou University Medical College. The trial protocol has previously been published (8). A prospective phase III clinical trial called “simultaneous modulated accelerated radiotherapy combined with chemotherapy vs concurrent chemoradiotherapy for esophageal cancer” is currently enrolling patients. Inclusion criteria: (a)Measurable lesions on imaging; (b)No obvious esophageal mass or lymph node enlargement compressing the esophagus on CT imaging after treatment; (c)No recurrence in the tumor area during follow-up ≥ 6 months. Exclusion criteria: (a)Control failure of the tumor area during or after treatment; (b)Failure to complete radiotherapy; (c)Surgery after complete radiotherapy. As shown in **Figure 1**, the final study enrolled 65 patients. These patients were divided into a training group (n= 43) and a validation group (n= 22) in a ratio of 2:1.

### Pre-treatment evaluation

The extent of the disease was evaluated by imaging, serological examination, and endoscopic biopsy. The clinical stage was defined according to the American Joint Cancer Committee (AJCC) staging system 6^th^ (28). LLT and ECI were evaluated by barium esophagography, endoscopy and CE-CT images (CT scanner: 16-row Spiral CT of Bright Speed Series of GE Medical Systems, USA). CT scanning parameters were setting as follows: Tube voltage,120KpV; Rotation time, 0.75 seconds; Pitch, 1.375; Matrix, 512×512; Field of visual, 360 mm×360 mm. The wall thickness was defined by measuring the thickest portion of the tumor. The extent of circumference involvement was demarcated as follows: (a)Level 1, ≤1/2 of circumference involvement; (b)Level 2, ≥1/2 of circumference involvement but less than whole circumference involvement; (c)Level 3, whole circumference involvement. Target area delineation and radiotherapy plans were determined by CT images analyzed in the Eclipse planning system.

### Treatment

All patients were treated with SIB in conjunction with chemotherapy. A higher-than-standard dose of 66 Gy/30 F was delivered to the gross tumor volume, and a lower dose of 54 Gy/30 F was delivered to the sub-clinical tumor volume. Chemotherapy was based on cisplatin and 5-fluorouracil (5-FU) for four cycles: two cycles of concurrent chemotherapy, and two cycles of adjuvant chemotherapy, after completing radiotherapy.

### Follow up

For the first 2 years after treatment, patients were assessed every 3 months and then twice a year. An evaluation of patients’ history, physical examination, serological test, chest X-ray with barium esophagography or CE-CT scan, and abdominal ultrasound were performed.

### Outcome indicators

**Figure 2:**
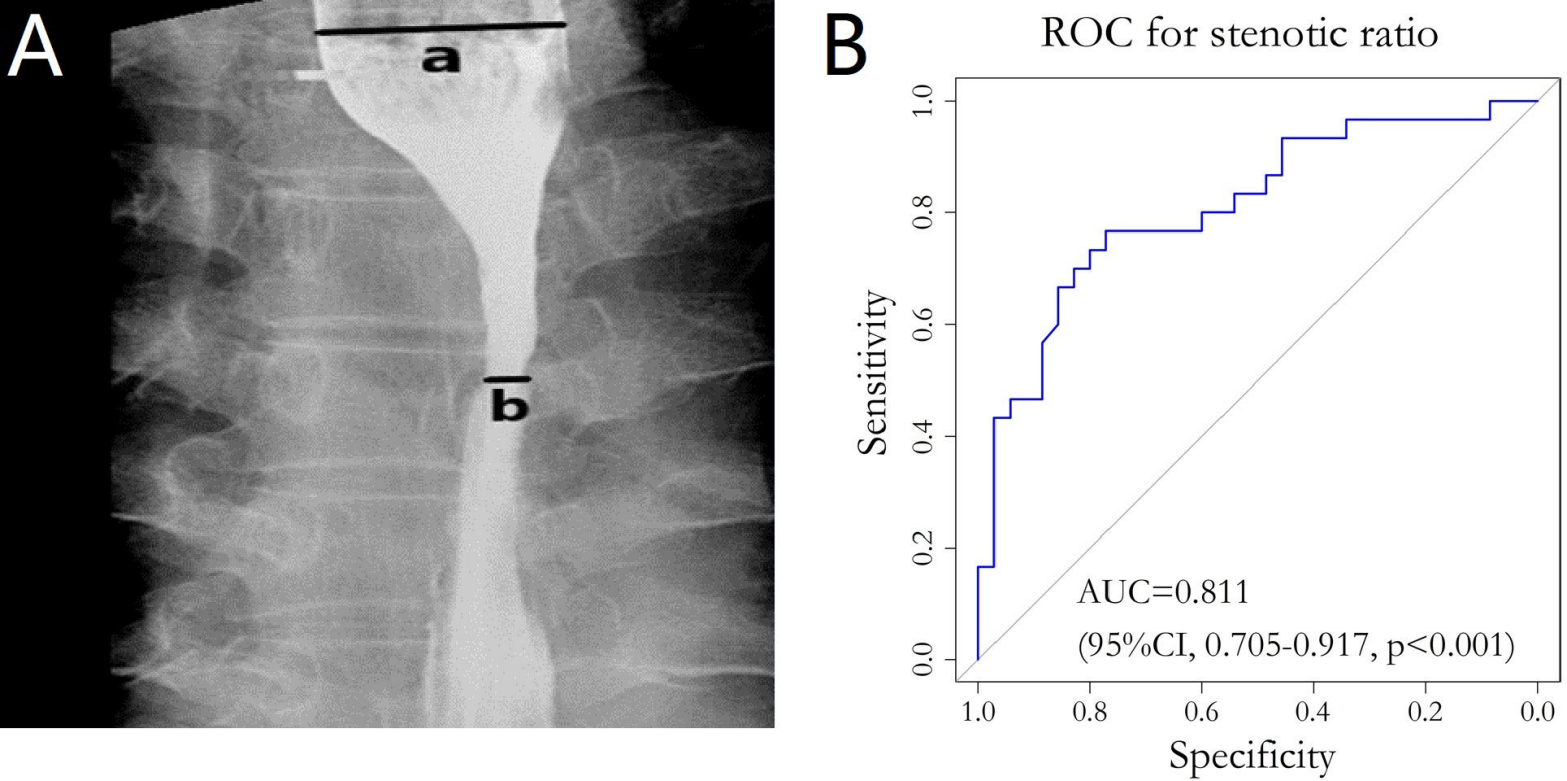
**(A)** Barium esophagography image. The widest part (line a in panel **A**) of the oral side and the narrowest part (line b in panel **A**) of the primary site were clearly demonstrated. **(B)** ROC curve for stenotic ratio and the diet group. ROC, Receiver operating Characteristic; AUC, Area under the curve.

Esophagograms, which were performed prior to, during, after, and during follow-up examination, were used to measure the degree of esophageal stenosis. By using a barium esophagogram, we measured the widest part (a in **Figure 2A**) of the oral side lumen diameter and the narrowest part (b in **Figure 2A**) of the primary site. The stenotic ratio (c; expressed as a percentage) was then determined as c = (a-b)/a×100% (12–14). The maximum value of the stenotic ratios in follow-up review at all points in time was defined as the degree of post-treatment BES.

In the follow up review, the severity of eating disorders was recorded and categorized according to the Radiation Therapy Oncology Group (RTOG) late radiation injury score: (a)Grade 0, none; (b)Grade 1, slightly difficulty in swallowing solids; (c)Grade 2, inability to swallow solid food normally, swallowing semi-solid food; (d)Grade3, ability to swallow only liquids; (e)Grade 4, necrosis or perforation fistula (29).

We integrated the severity of eating disorders Grade 0–1 into the normal diet group and Grade≥2 into the non-normal diet group, then combined the stenotic ratio with the diet grouping to plot the ROC curve. The AUC was calculated to quantify the accuracy of the stenotic ratio in assessing the degree of esophageal stenosis. The optimal cut-off value of the stenotic ratio was determined according to the Youden index. For univariate analysis, a chi-square test, t-test, and rank sum test were performed to explore the correlation between stenotic ratio and clinical factors. For multivariate analysis, binary logistic regression analysis and a linear regression model were used.

### CE-CT image acquisition and radiomics extraction

All patients underwent pre-treatment CE-CT scans (Philips Brilliance CT Big Bore Oncology Configuration, Cleveland, OH, USA). The CT voxel size was 1.0 × 1.0 × 3.0 mm^3^. The CT images were transmitted to the radiation therapy planning system (Eclipse Planning System version 10.0) *via* the DICOM 3.0 port. All gross tumor volumes (GTVs) were delineated on the planning CT scans by experienced radiation oncologists. The radiomics features were extracted from every GTV using MATLAB R2016a (Mathworks, Natick, USA) and its toolbox (https://cn.mathworks.com/). These features included four groups: the intensity features, the geometric features and the texture features. According to the first-order statistics, the intensity features were calculated from the histogram of voxel intensity values in the volume of interest (VOI). The geometric features describe the shape of the VOI (30). The texture features calculated in all three-dimensional directions within the VOI, which can quantify intra-tumor heterogeneity differences, consist of gray level co-occurrence matrix (GLCM), neighborhood grey-tone difference matrix (NGTDM), gray level size zone matrix (GLSZM) and gray level run length matrix (GLRLM) (31–34). Overall, 96 radiomic features were extracted from every GTV. The specific types and algorithms for radiomic feature extraction have been discussed in previous studies (35, 36).

### Radiomics features selection and model development

Univariate analysis was used to evaluate radiomics factors for BES. Radiomics variables with a p-value >0.250 were excluded from further analysis. Pearson correlation analysis was used to reduce the correlations between radiomics features. For example, for a pair of features with high correlation (i.e., the absolute value of correlation coefficient ≥ 0.8), the one with a lower p-value in the univariate analysis remained. The least absolute shrinkage and selection operator (LASSO) was chosen for the logistic regression model to select the most useful predictive features and create a radiomics signature model (defined as the Rad-score).

### Clinical features selection and model development

Before starting treatment, clinical data including gender, age, tumor location and the TNM stage was gathered. LLT, ECI and number of CT layers in which the wall thickness of affected esophagus (NEWT) >1cm were collected from barium esophagography, endoscopy and CE-CT images. Univariate and multivariate analysis were used to identify the clinical factors correlating with BES after SIB. The potential clinical risk factors constituted the clinical model.

### Combined model development

The individualized prediction model included potential clinical risk factors and the Rad-score using multivariate logistic regression analysis. To visualize the patient-level probability estimate of BES, a nomogram was developed based on multivariate logistic regression analysis and tested in the validation cohort.

### Assessment of the Rad-score and nomogram

Because the clinical factors (such as ECI, LLT and NEWT>1cm) were measured on the CE-CT imaging, the VIF analysis was also used to assess the collinearity information among the clinical factors and final radiomics features. Variance inflation factor (VIF) was used to evaluate the collinearity among the final radiomics features that constituted the Rad-score. The performance of each model was evaluated using the area under the receiver operating characteristic curves (AUCs), accuracy, sensitivity and specificity.

The predictive power of the nomogram was quantified using Harrell’s concordance index (C-index) and assessed using the calibration curve. The Hosmer-Lemeshow test was used to assess the goodness-of-fit of the nomogram (37). Decision curve analysis (DCA) was used to quantify the net benefit at different threshold probabilities and determine the clinical usefulness of the nomogram.

### Statistical analysis

For univariate analysis, a chi-square test, t-test, and rank sum test were performed to explore the correlation between stenotic ratio and clinical factors. For multivariate analysis, binary logistic regression analysis and a linear regression model were used.

All statistical tests were conducted using R software version 4.0.5 and SPSS (version 23.0; IBM Corp., Armonk, NY, USA). The “glmnet” package was used to analyze the LASSO logistic model. The “pROC” and “car” were used to calculate the ROC curves and VIF. The C-index was calculated using the Kaplan–Meier “survival” package. The nomogram and calibration curve were built by using “rms” package. The Hosmer-Lemeshow test was calculated using the “generalhoslem” package in the R environment. Differences were considered statistically significant at p < 0.05.

## Result

### Patients’ characteristics

The patient characteristics for the two cohorts are shown in **Table 1**. The average age of 65 patients was 61.16 ± 5.77. There were no differences in patient characteristics between the training group and validation group.

**Table 1:** Clinical characteristics of 65 patients with ESCC after definitive CCRT.

Factors	Training cohort n (%)	Validation cohort n (%)	p-value
Age, years			0.624^b^
Average ± SD	61.16 ± 5.77	61.95 ± 6.78	
BMI, Kg/m^2^			0.330^b^
Average ± SD	21.52 ± 3.31	20.70 ± 2.87	
Gender			0.906^c^
Male Female	28 (65.1%) 15 (34.9%)	14 (63.6%) 8 (36.4%)	
Tumour location			0.671^c^
Cervical Upper Middle	3 (7.0%) 18 (41.9%) 22 (51.2%)	3 (13.6%) 9 (40.9%) 10 (45.5%)	
T stage^a^			0.966^c^
T2 T3 T4	9 (20.9%) 21 (48.8%) 13 (30.2%)	5 (22.7%) 11 (50.0%) 6 (27.3%)	
N stage^a^			0.335^c^
N0 N1	19 (44.2%) 24 (55.8%)	7 (40.0%) 15 (68.2%)	
M stage^a^			0.572^c^
M0 M1	37 (86.0%) 6 (14.0%)	20 (90.9%) 2 (9.1%)	
Clinical stage^a^			0.776^c^
II stage III stage IV stage	17 (39.5%) 20 (46.5%) 6 (14.0%)	8 (36.4%) 12 (54.5%) 2 (9.1%)	
ECI			0.778
Level 1	2	2	
Level 2	27	13	
Level 3	14	17	
LLT			0.430
Average ± SD	5.01 ± 1.78	5.06 ± 1.51	
NEWT>1cm			0.911
Average± SD	9.65 ± 7.34	11.14 ± 6.72	

ESCC, esophageal squamous cell carcinoma; CCRT, concurrent chemoradiotherapy; AJCC, American Joint Committee on Cancer staging system (version 6.0^th^); RT, radiotherapy; PF, cisplatin and 5-fluorouracil.

a American Joint Committee on Cancer (AJCC) staging system (version 6.0^th^)

b p-value was analysed using the independent samples t-test

c p-value was analysed using the chi-squared test.

IBM, imaging biomarker; CI, confidence interval; HR, hazard ratio.

### Benign esophageal stenosis after treatment

The last date of follow-up was December 22, 2019, and the median follow-up period was 62 months (17-82 months) for all patients. The change in the mean esophageal stenotic ratio of 65 patients before treatment to 1 year after treatment is shown in **Table S1** and **Figure S1**. The change in the mean esophageal stenotic ratio of 48 patients (17 eliminated, 1 for recurrence, 2 for death, and 14 for missing follow-up) from 3 to 18 months after treatment are shown in **Table S2** and **Figure S2**. It tended to decrease with time and reached a plateau in the ninth months after treatment.

The peak stenotic ratio for 24 (36.9%), 20 (30.8%), 11 (16.9%), 6 (9.2%), 2 (3.1%), 1 (1.5%), and 1 (1.5%) patient(s) occurred in the third, sixth, ninth, twelfth, fifteenth, eighteenth, and twenty-first months after treatment, respectively. Thirty-five patients (53.8%) had a normal diet when they had a peak stenotic ratio, 26 (40%) had a semi-solid diet, and 4 (6.2%) had a liquid diet. We divided these patients into a normal diet group (35 patients, 53.8%) and a non-normal diet group (30 patients, 46.2%) according to the RTOG late radiation injury score. The ROC curve was plotted by considering the stenotic ratio as the test variable and the diet group as the status variable, which resulted in AUC=0.811 (95% CI: 0.705-0.917, p<0.001) (**Figure 2B**). The optimal cut-off value for stenotic ratio was determined to be 58.2% according to the Youden index, and 31 cases (47.7%) with a stenotic ratio >58.2% were defined as the benign stenotic group (high risk group). The rest of patients were defined as the low risk group.

### Radiomics selection and Rad-score constructing

**Figure 3:**
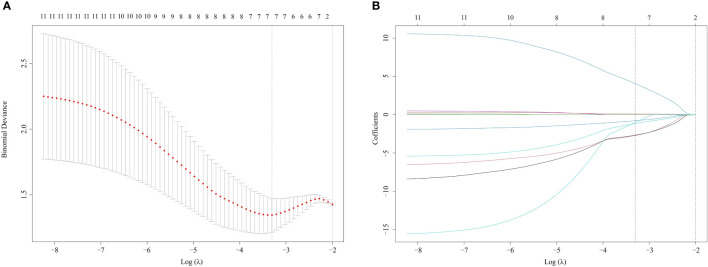
Radiomics selection using LASSO logistic regression model **(A)**. The tuning parameter λ selection of LASSO model with 10-fold cross-validation was performed to select radiomic features. At the optimal tuning parameter λ value of 0.036 and log (λ) = -3.322, the left dotted vertical line was set with the minimum criteria where 7 radiomic features were selected. **(B)**. LASSO coefficient profile of 11 Radiomic features. A coefficient profile plot was generated against the log (λ) sequence. The dotted vertical lines were drawn at the 6 non-zero coefficients, with the optimal value of λ. LASSO, least absolute shrinkage and selection operator.

Ninety-six radiomics features were reduced to 30 potential factors, which had a p-value ≤ 0.25. Nineteen features were excluded after comparing the inter-variable Pearson correlation analysis. The remained 11 features were performed with non-zero coefficients in the LASSO logistic regression model. Ultimately 7 of them were chosen to construct the Rad-score. As shown in **Figure 3**, with the optimal tuning parameter λ value of 0.036 and log (λ) = -3.322, the Rad-score calculation formula was constructed using the LASSO logistic regression model (Formula 1):

Rad-score = −0.0037 × Max + 4.7716 × SphericalDisproportion − 1.4654 × Idistcent−2.9222 × Informaiton Measure of Correlation2_GLCM+ 0.1131 × Run_Percentage _GLRLM−0.9177 × Texture_Strength_NGTDM−1.7964 × Small_Zone_Emphasis_GLSZM + 5

A constant value 5 was used to obtain a Rad-score >0 from the calculation formula. The VIFs of the seven radiomics features were tolerable, ranging from 1.336-2.341 (**Table S3**).

### Development of individualized prediction model

Clinical factors were analyzed using univariate and multivariate Logistic regression, as shown in **Table 2**. In the training cohort, factors showing a significant correlation with BES after treatment were ECI (p=0.027), LLT (p=0.097), and the number of CT layers in which the wall thickness of affected esophagus>1cm (NEWT>1cm) (p=0.028) in the univariate analysis. VIFs of the seven radiomics features and the three clinical factors were tolerable (VIF<10), ranging from 1.593-8.640 (**Table S3**). We combined the clinical characteristics and the Rad-score into a multivariate logistic regression model.

**Table 2:** Univariate and multivariate association of Rad-score and clinical characteristic Logistic regression analysis of BES (likelihood Ratio: Backward stepwise).

Variables	Training cohort				Validation cohort			
	Univariate		Multivriate		Univariate		Multivriate	
	OR	*p*	OR(95%CI)	*p*	OR	*p*	OR(95%CI)	*p*
Age	1.010	0.848						
Gender	1.319	0.666						
Tumour location	1.524	0.401						
Clinical stage	0.722	0.470						
BMI	1.035	0.712						
LLT	1.365	0.097	1.008 (0.571-1.780)	0.977	1.614	0.148	0.566(0.165-1.945)	0.367
ECI	4.314	0.027	3.112(0.777-12.469)	0.109	5.210	0.069	6.080(0.735-50.303)	0.094
NEWT>1cm	1.116	0.028	1.087 (0.981-1.206)	0.112	1.249	0.027	1.384(1.008-1.901)	0.044

OR, odds ratio; CI, confidence interval; BMI, Body Mass Index; LLT, longitudinal length of tumor; ECI, the extent of circumference involvement; NEWT>1cm, the number of CT layers in which the wall thickness of affected esophagus>1cm.

The bold values refers to the clinical factors which were included in the clinical models.

### Performance of the model and nomogram

**Figure 4:**
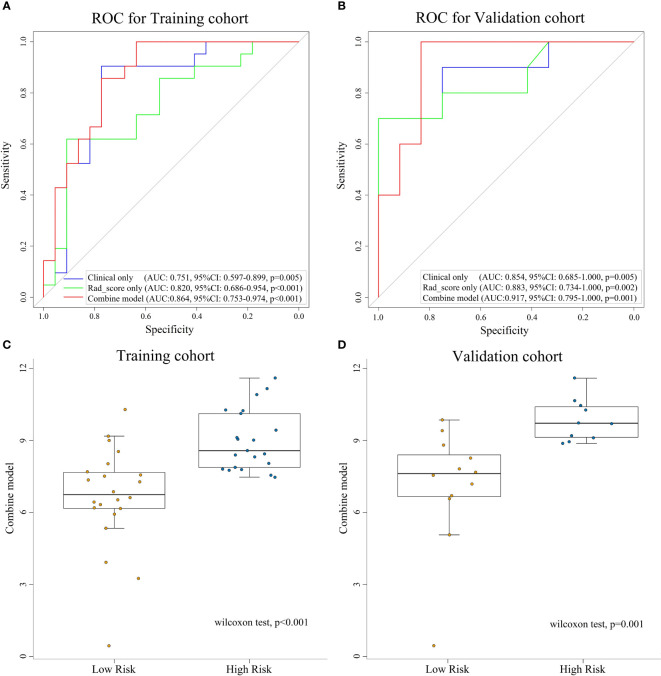
ROC curves for assessing the different performance of three models in training cohort **(A)** and validation cohort **(B)**. The box plots of combine model for low-risk and high-risk groups for training cohort **(C)** and validation cohort **(D)**. ROC, receive operation characteristic; AUC, area under the curve; CI, confidence interval; Rad-score, radiomic score.

**Figure 5:**
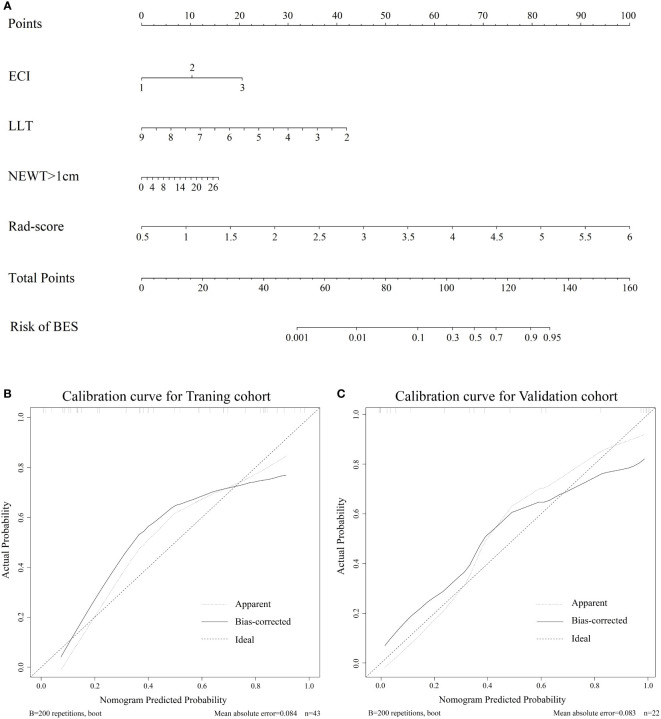
**(A)**. The nomogram for the prediction of BES. The constructed nomograms were used to estimate the risk of BES for individual ESCC patients. Calibration curves of the combined nomogram in the training cohort **(B)** and validation cohort **(C)**. The calibration curves describe the calibration of the combine nomogram in terms of the conformity between the predicted risk of BES and observed BES outcomes. The 45° dotted line represents a perfect prediction, the solid lines represent the bias-corrected performance of the combine nomogram. BES, benign esophageal stenosis; ESCC, esophageal squamous cell carcinoma; ECI: the extent of circumference involvement; LLT: longitudinal length of tumor; NEWT>1cm: number of CT layers with esophageal wall thickness >1 cm.

**Figure 6:**
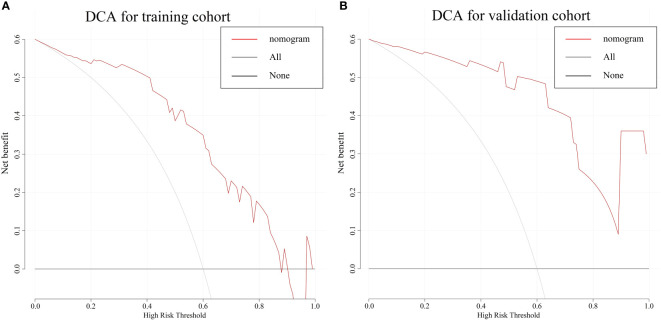
The DCA for the combine nomogram in the training cohort **(A)** and validation cohort **(B)**. The y-axis represents the net benefit. The x-axis represents the threshold probability. The red line represents the radiomics nomogram. The grey line represents the hypothesis that all patients had BES. The black line represents the hypothesis that no patients had BES. The DCA in two cohorts showed the nomogram had clinical benefit. DCA, Decision curve analysis; BES, benign esophageal stenosis.

The combine model performed the best among three models. **Figures 4A, B** deplicted the AUCs of different models. For BES, the Rad-score model was superior to the clinical model. The combine model outperformed the Rad-score or the clinical model in the training cohort, and the results were replicated in the validation cohort. The box plot method was used to compare low-risk and high-risk patients in the BES as shown in **Figures 4C, D**. And the results revealed significant differences (p<0.05, wilcoxon test) between two subgroups of BES in two cohorts. We also constructed a nomogram to visualize the logistic regression model of BES (**Figures 5A**). As shown in **Figure 5B, C**, the calibration curve of the nomogram for the probability prediction of BES had good prognostic performance. The DCA showed the nomogram had clinical utility in predicting the power of risk of BES within a wide range of reasonable threshold probability (**Figure 6**). The Hosmer-Lemeshow test revealed no deviation from model fitting for the training cohort (p=0.451) and validation cohort (p=0.481). The C-indices of the nomogram were 0.864 and 0.958 for the training and validation cohorts, respectively.

## Discussion

For patients with locally advanced EC, approximately half had local recurrence and poor overall survival (38, 39). Some researchers have applied SIB to the therapy of EC to improve local control (8). Nonetheless, information on late toxicity of SIB, which contributes to the comprehensive evaluation of this therapeutic mode, is limited. Benign esophageal stenosis is one of the most common late toxicities that leads to significant deterioration in quality of life despite tumor regression. The benefits must be balanced against the risk of toxicities. Thus, developing an individual predictive model is critical for clinical decision making.

We expected that characteristics other than clinical factors, such as texture and distribution on CT imaging, might contribute to the severity of benign esophageal stenosis. As a result, our study yields promising results. We found that the Rad-score, which consisted of seven radiomics features, was discovered to be an independent risk factor for BES after SIB. In terms of clinical factors, CEI, LLT and NEWT>1cm were identified as potential factors for BES in univariate analysis, however these clinical factors were only weakly correlated with BES in multivariate analysis.

Although several studies have identified risk factors for esophageal stenosis, clinicians are unable to identify specific patients who may develop BES after radiotherapy (12–14). It is necessary to identify patients who have a high risk of BES after SIB before treatment, as these patients might be more suitable for surgery or immunotherapy. And the radiation dose should be further explored in such patients. Hence, a prediction model based on radiomics and clinical factors that discriminated severe BES after SIB with high diagnostic performance was developed in this study.

The radiomics signature model incorporates some individual radiomics features as predictors to probe the clinical utility of features that have been explored and investigated in many studies (40, 41). Max was extracted from the intensity features. It measures the maximum value of the gray level intensity. According to our findings, a smaller Max value may be related to a poor BES result. SphericalDisproportion refers to the ratio of the tumor region’s perimeter to the perimeter of a sphere with the same surface area as the tumor region. Idistcent describes the maximum distance between the vertices of the tumor surface grid in the axial plane. Tumors with a larger SphericalDisproportion or a smaller Idistcent had more irregular shape, which was associated with poorer treatment response (15, 42, 43). In terms of texture feature, the Information Measure of Correlation2_GLCM refers to the consistency of the gray level of image texture in the row or column directions. It is high when gray levels are equally distributed along the row or column direction in contoured structures. A smaller Information Measure of Correlation2_GLCM, indicating the ROI heterogeneity (44). Run_Percentage_GLRLM measures the texture roughness by dividing run length by voxels in ROI. Texture_Strength_NGTDM represents the significance and uniqueness of voxels on a three-dimensional level. Small_Zone_Emphasis_GLSZM describes of the distribution of small areas. The finer the contoured structures are, the larger the value, the smaller the zone. The previous studies observed that Information Measure of Correlation2_GLCM, the Run_Percentage_GLRLM, Texture_Strength_NGTDM and Small_Zone_Emphasis_GLSZM are highly relevant to the heterogeneity and prognosis of Specific types of tumor (15, 44, 45). These results demonstrate the possibility of using radiomics multivariate analysis and the high OR of the radiomics signature model might able to predict BES in patients with ESCC. However, these seven radiomic features did not achieve the significant statistical value due to insufficient number of patients. It needs to be further confirmed in the validation cohort and larger prospective cohorts. Several previous studies have reported that combining radiomic signatures (or features) and clinical risk factors improved the predictive accuracy of these models (26, 35, 46, 47). Thus, we developed a nomogram that incorporates the Rad-score as well as these clinical factors. These clinical factors are generally available during treatment, and the collection of information does not require additional examinations or place an additional economic burden on patients. Despite the fact that these clinical factors were insignificantly different in multivariate analysis, incorporating them into the radiomics signature model, which comprised the combined model, improved the AUC in predicting BES and achieved excellent discrimination in this cohort.

This study demonstrated that the esophageal stenotic ratio tended to decrease during and after treatment, with the mean stenotic ratio dropping from 72.0% before treatment to 46.5% 1 year after treatment, reflecting a distinct remission of stenosis caused by tumor mass. Re-stenosis was considered because the maximum stenotic ratio during follow-up was greater than that at the completion of treatment. Two other studies (12, 14) reported that the peak stenotic ratio occurred–5-8 and 6-8 months after treatment, but specific numbers and proportions of cases at each point in time were not displayed. In previous studies (12–14), the evaluation of stenosis has basically referred to the barium esophagogram as follows: Grade 1 (<25%), Grade 2 (25~50%), Grade 3 (50~75%), and Grade 4 (75~100%); grade ≥3 is defined as stenosis, or referred to the RTOG late radiation injury score, which was classified based on the patients’ subjective experience of eating disorders. Atsumi K et al., Wang et al., and Luo et al. all used stenotic ratio to assess the degree of BES after high dose radiotherapy (ranging 54-71.4Gy, 56-66Gy, 56-70Gy, respectively), and 23%, 43.5% and 33.8% of patients had a stenotic ratio >50% respectively in their researches (12–14). All the above studies found BES was not correlated with RT dose. In this study, we combined the RTOG late radiation injury score and stenotic ratio to create an ROC curve, and the result showed AUC=0.811, indicating that the stenotic ratio was considered capable of objectively evaluating the extent of esophageal stenosis. According to the optimal cut-off value, stenotic ratio>58.2% was defined as benign stenosis. The rate of BES after 66 Gy radiotherapy was 47.7%. The evaluation of stenosis could be more accurate and convincing by the integration of the two above.

Previous studies have explored the risk factors for esophageal stenosis after conventional fractional radiotherapy. The extent of circumference involvement, T stage, tumor length, and the wall thickness of the affected esophagus has been confirmed by Atsumi et al. in a 109 EC patient study, Wang et al. in a 61-patient study, Luo et al. in a 71-patient study, and Kim et al. in a 62-patient study (12–14, 48). The extent of circumference involvement was a risk factor for stenosis after endoscopic mucosal resection of early EC (49).

Dysphagia is the most common symptom in advanced patients and has a significant impact on quality of life. The mechanism of BES after chemoradiotherapy has not been determined due to limited pathological data. It is commonly assumed that post-radiotherapy esophageal stenosis is caused by radiation-induced fibrosis (RIF). Because of fibrosis and inflammation of the submucosa and muscular layers, the esophagus loses its elasticity, resulting in post-radiotherapy esophageal stenosis 4-12 months after therapy and developing in a few years (50). However, the stenotic ratio of patients in our study tended to reach a plateau after 9 months. This may be because long-term esophageal peristalsis controlled by autonomic nerves, even without food intake, may decrease the damage of fibrosis. For BES, balloon dilatation, stent implantation, bypass operation, and drug infusion are the most commonly used palliative treatments (51), but they had unsatisfactory outcomes for high re-stenosis rate and complications, like perforation and hemorrhage (52–54).

This study was conducted prospectively to indentify the risk factors associated with BES by combining objective evaluation using esophagography with subjective evaluation of the severity of eating disorders in patients, and build an individual predictive model incorporating radiomics features and clinical factors. It could be a useful guide when choosing a treatment option for patients with EC as well as an important piece of information when acquiring a patient’s informed consent before radiotherapy.

The innovation of this study is that this study was a prospective study for BES after SIB for ESCC. Since the difficulty of long time follow up, there is scarce data for BES after high dose radiotherapy for esophageal cancer. The application of radiomics in CE-CT imaging has been focused on specific topics, such as survival outcomes and diagnosis (55). To the best of our knowledge, this is the first predict model that applied radiomics to assess BES after SIB.

The limitations of this study include the lack of comparison with patients receiving standard-dose radiotherapy because all the patients in these prospective studies received SIB. The NCCN Guidelines state that a standard dose of definitive radiation for esophageal cancer is 50–50,4 Gy (1.8–2.0 Gy/day) (total 25–28 fractions). However, more than 50% of patients who had standard-dose CRT subsequently experienced recurrence or distant metastases and passed away from this illness (56). A dose of 60.0 Gy or more has become a more common dose of CCRT in Asian nations where ESCC is the predominate histological type since studies have shown that a greater dose than 50.4 Gy of CCRT could be safely administered without significant adverse effects and yield a high probability of local control (8, 57, 58). Since high dose radiotherapy of 60 Gy or more with 2 Gy per fraction, is frequently used in China to treat esophageal cancer, it can be challenging to collect clinical information concerning standard dose of radiotherapy, particularly for a prospective study. Our hospital is currently conducting a phase III trial, and we will eventually include a standard dose of radiotherapy in our prediction model. There were no T1 patients in our study; therefore, the impact of T stage on esophageal stenosis after SIB needs further verification. Another limitation of our study is the small sample size and the lack of external validation of the model. Clinical data for BES was difficult to acquire due to long time follow up. Wang et al. analyzed BES after radiotherapy ranging 56-66Gy and included 62 patients from 2005 to 2008 (12). Luo et al. analyzed BES after radiotherapy ranging 56-70Gy and included 71 patients from 2010 to 2013 (14). Jun W. Kim et al. analyzed BES after radiotherapy ranging 45-90Gy and included 62 patients from 2001 to 2015 (48). These were all retrospective studies with a wider range of radiotherapy dose. The challenge of gathering information of BES is a pervasive problem in this research field. That is why the sample size in this study is small and it is indeed a weakness. To overcome the shortcoming of limited sample size we did use the 10-fold cross validation method in this study. K-fold cross validation was reported to reduce the uncertainty of input dataset partition in previous study (59, 60). Multicenter validation with a larger sample size is required for clinical applications.

## Conclusion

In conclusion, BES due to tumor mass could achieve varying degrees of remission after simultaneous modulated accelerated radiotherapy, but BES occurs after radiotherapy at the same time. BES after SIB was potential associated with the Rad-score, CEI, LLT and NEWT>1cm. We developed a nomogram that incorporates both the Rad-score and clinical prognostic factors to predict the risk of BES in patients with ESCC who received definitive CCRT.

## Data availability statement

The raw data supporting the conclusions of this article will be made available by the authors, without undue reservation.

## Ethics statement

This study was performed in line with the principles of the Declaration of Helsinki. Approval was granted by the Ethics Committee of Shantou University Medical College (2013-04-28/No. SUMC2013XM-0085). Informed consent was obtained from all individual participants included in the study. The patients/participants provided their written informed consent to participate in this study.

## Author contributions

All authors contributed to the study conception and design. Material preparation, data collection and analysis were performed by CC, WL, CZ, and SW. The first draft of the manuscript was written by WL and all authors commented on previous versions of the manuscript. All authors contributed to the article and approved the submitted version.

## Supplementary material

Supplementary Figure 1 The change in the mean esophageal stenotic ratio of 65 patients before treatment to 1 year after treatment. Seven points in x axis represent before treatment, twentieth fraction, complete treatment, 3, 6, 9, and 12 months after completing treatment.

Supplementary Figure 2 The change in the mean esophageal stenotic ratio of 48 patients from 3 to 18 months after treatment. Six points in x axis represent 3, 6, 9, 12, 15, and 18 months after completing treatment.

## References

[1] Lin Y Totsuka Y Shan B Wang C Wei W Qiao Y. Esophageal cancer in high-risk areas of China: Research progress and challenges. Ann Epidemiol (2017) 27(3):215–21. doi: 10.1016/j.annepidem.2016.11.004 28007352

[2] Feng RM Zong YN Cao SM Xu RH. Current cancer situation in China: good or bad news from the 2018 global cancer statistics? Cancer Commun (London England) (2019) 39(1):22. doi: 10.1186/s40880-019-0368-6 PMC6487510 31030667

[3] Doosti-Irani A Holakouie-Naieni K Rahimi-Foroushani A Mansournia MA Haddad P. A network meta-analysis of the treatments for esophageal squamous cell carcinoma in terms of survival. Crit Rev Oncol Hematol (2018) 127:80–90. doi: 10.1016/j.critrevonc.2018.05.007 29891115

[4] Cooper JS Guo MD Herskovic A Macdonald JS Martenson JA Jr. Al-Sarraf M. Chemoradiotherapy of locally advanced esophageal cancer: Long-term follow-up of a prospective randomized trial (RTOG 85-01). Radiat Ther Oncol Group Jama (1999) 281(17):1623–7. doi: 10.1001/jama.281.17.1623 10235156

[5] Kim HJ Suh YG Lee YC Lee SK Shin SK Cho BC. Dose-response relationship between radiation dose and loco-regional control in patients with stage II-III esophageal cancer treated with definitive chemoradiotherapy. Cancer Res Treat Off J Korean Cancer Assoc (2017) 49(3):669–77. doi: 10.4143/crt.2016.354 PMC5512369 27737537

[6] Yu WW Zhu ZF Fu XL Zhao KL Mao JF Wu KL. Simultaneous integrated boost intensity-modulated radiotherapy in esophageal carcinoma: Early results of a phase II study. Strahlentherapie und Onkologie: Organ der Deutschen Rontgengesellschaft [et al] (2014) 190(11):979–86. doi: 10.1007/s00066-014-0636-y 24609941

[7] Li C Ni W Wang X Zhou Z Deng W Chang X. A phase I/II radiation dose escalation trial using simultaneous integrated boost technique with elective nodal irradiation and concurrent chemotherapy for unresectable esophageal cancer. Radiat Oncol (London England) (2019) 14(1):48. doi: 10.1186/s13014-019-1249-5 PMC6420772 30876442

[8] Chen J Guo H Zhai T Chang D Chen Z Huang R. Radiation dose escalation by simultaneous modulated accelerated radiotherapy combined with chemotherapy for esophageal cancer: a phase II study. Oncotarget (2016) 7(16):22711–9. doi: 10.18632/oncotarget.8050 PMC5008394 26992206

[9] Shah JN. Benign refractory esophageal strictures: widening the endoscopist's role. Gastrointest Endosc (2006) 63(1):164–7. doi: 10.1016/j.gie.2005.08.033 16377341

[10] Coia LR Engstrom PF Paul A. Nonsurgical management of esophageal cancer: report of a study of combined radiotherapy and chemotherapy. J Clin oncology: Off J Am Soc Clin Oncol (1987) 5(11):1783–90. doi: 10.1200/JCO.1987.5.11.1783 2445931

[11] Coia LR Myerson RJ Tepper JE. Late effects of radiation therapy on the gastrointestinal tract. Int J Radiat Oncol Biol Phys (1995) 31(5):1213–36. doi: 10.1016/0360-3016(94)00419-L 7713784

[12] Wang C Lv C Wang J Liu J Guo J Li H. Analysis of risk factors of developing esophageal stricture after radiotherapy for esophageal carcinoma. Zhong Guo Zhong Liu Lin Chuang (2010) 37(10):579–81+86. doi: 10.3969/j.issn.1000-8179.2010.10.011

[13] Atsumi K Shioyama Y Arimura H Terashima K Matsuki T Ohga S. Esophageal stenosis associated with tumor regression in radiotherapy for esophageal cancer: frequency and prediction. Int J Radiat Oncol Biol Phys (2012) 82(5):1973–80. doi: 10.1016/j.ijrobp.2011.01.047 21477944

[14] Luo J Lu X Zhou X Lu Z. Shi guan ai fang liao hou liang xing xia zhai de xiang guan yin su fen xi [ analysis of risk factors of esophageal benign stricture after radiotherapy for esophageal carcinoma]. Zhong Hua Zhong Liu Fang Zhi Za Zhi (2015) 22(13):1046–9. doi: 10.16073/j.cnki.cjcpt.2015.13.012

[15] Lambin P Leijenaar RTH Deist TM Peerlings J de Jong EEC van Timmeren J. Radiomics: the bridge between medical imaging and personalized medicine. Nat Rev Clin Oncol (2017) 14(12):749–62. doi: 10.1038/nrclinonc.2017.141 28975929

[16] Thawani R McLane M Beig N Ghose S Prasanna P Velcheti V. Radiomics and radiogenomics in lung cancer: A review for the clinician. Lung Cancer (Amsterdam Netherlands) (2018) 115:34–41. doi: 10.1016/j.lungcan.2017.10.015 29290259

[17] Bodalal Z Trebeschi S Nguyen-Kim TDL Schats W Beets-Tan R. Radiogenomics: bridging imaging and genomics. Abdominal Radiol (New York) (2019) 44(6):1960–84. doi: 10.1007/s00261-019-02028-w 31049614

[18] Gillies RJ Kinahan PE Hricak H. Radiomics: Images are more than pictures, they are data. Radiology (2016) 278(2):563–77. doi: 10.1148/radiol.2015151169 PMC4734157 26579733

[19] van Rossum PSN Xu C Fried DV Goense L Court LE Lin SH. The emerging field of radiomics in esophageal cancer: current evidence and future potential. Trans Cancer Res (2016) 5(4):410–23. doi: 10.21037/tcr.2016.06.19 PMC6343849 30687593

[20] Limkin EJ Sun R Dercle L Zacharaki EI Robert C Reuzé S. Promises and challenges for the implementation of computational medical imaging (radiomics) in oncology. Ann Oncol Off J Eur Soc Med Oncol (2017) 28(6):1191–206. doi: 10.1093/annonc/mdx034 28168275

[21] Sah BR Owczarczyk K Siddique M Cook GJR Goh V. Radiomics in esophageal and gastric cancer. Abdominal Radiol (New York) (2019) 44(6):2048–58. doi: 10.1007/s00261-018-1724-8 PMC6934409 30116873

[22] Liu L Yi X Lu C Qi L Zhang Y Li M. Applications of radiomics in genitourinary tumors. Am J Cancer Res (2020) 10(8):2293–308. PMC7471369 32905456

[23] Sjoquist KM Burmeister BH Smithers BM Zalcberg JR Simes RJ Barbour A. Survival after neoadjuvant chemotherapy or chemoradiotherapy for resectable oesophageal carcinoma: An updated meta-analysis. Lancet Oncol (2011) 12(7):681–92. doi: 10.1016/S1470-2045(11)70142-5 21684205

[24] Cheng L Wu L Chen S Ye W Liu Z Liang C. [CT-based radiomics analysis for evaluating the differentiation degree of esophageal squamous carcinoma]. Zhong nan da xue xue bao Yi xue ban = J Cent South Univ Med Sci (2019) 44(3):251–6. doi: 10.11817/j.issn.1672-7347.2019.03.004 30971516

[25] Wu L Wang C Tan X Cheng Z Zhao K Yan L. Radiomics approach for preoperative identification of stages I-II and III-IV of esophageal cancer. Chin J Cancer Res = Chung-kuo yen cheng yen chiu (2018) 30(4):396–405. doi: 10.21147/j.issn.1000-9604.2018.04.02 30210219 PMC6129566

[26] Shen C Liu Z Wang Z Guo J Zhang H Wang Y. Building CT radiomics based nomogram for preoperative esophageal cancer patients lymph node metastasis prediction. Trans Oncol (2018) 11(3):815–24. doi: 10.1016/j.tranon.2018.04.005 PMC6154864 29727831

[27] Xie C Yang P Zhang X Xu L Wang X Li X. Sub-Region based radiomics analysis for survival prediction in oesophageal tumours treated by definitive concurrent chemoradiotherapy. EBioMedicine (2019) 44:289–97. doi: 10.1016/j.ebiom.2019.05.023 PMC6606893 31129097

[28] Greene FL Page DL Fleming ID Fritz A Balch CM Haller DG. AJCC cancer staging manual (6th edition). 6 edition. New York: Springer (2002). pp. 91–8.

[29] Cox JD Stetz J Pajak TF. Toxicity criteria of the radiation therapy oncology group (RTOG) and the European organization for research and treatment of cancer (EORTC). Int J Radiat oncology biology physics (1995) 31(5):1341–6. doi: 10.1016/0360-3016(95)00060-C 7713792

[30] Zwanenburg A Vallières M Abdalah MA Aerts HJWL Andrearczyk V Apte A. The image biomarker standardization initiative: Standardized quantitative radiomics for high-throughput image-based phenotyping. Radiology (2020) 295(2):328–38. doi: 10.1148/radiol.2020191145 PMC7193906 32154773

[31] Amadasun MaK R. Textural features corresponding to textural properties. IEEE Trans Systems Man Cybernetics (1989) 19:1264–74. doi: 10.1109/21.44046

[32] Sun C Wee WG. Neighboring gray level dependence matrix for texture classification. Comput Gr Image Process (1983) 23:341–52. doi: 10.1016/0734-189X(83)90032-4

[33] Thibault G Angulo J Meyer F. Advanced statistical matrices for texture characterization: Application to cell classification. IEEE Trans BioMed Eng (2014) 61(3):630–7. doi: 10.1109/TBME.2013.2284600 24108747

[34] Galloway MM. Texture analysis using gray level run lengths. Comput Graphics Image Process (1975) 4(2):172–9. doi: 10.1016/S0146-664X(75)80008-6

[35] Zhai TT van Dijk LV Huang BT Lin ZX Ribeiro CO Brouwer CL. Improving the prediction of overall survival for head and neck cancer patients using image biomarkers in combination with clinical parameters. Radiotherapy oncology: J Eur Soc Ther Radiol Oncol (2017) 124(2):256–62. doi: 10.1016/j.radonc.2017.07.013 28764926

[36] Zeng C Zhai T Chen J Guo L Huang B Guo H. Imaging biomarkers of contrast-enhanced computed tomography predict survival in oesophageal cancer after definitive concurrent chemoradiotherapy. Radiat Oncol (London England) (2021) 16(1):8. doi: 10.1186/s13014-020-01699-w PMC7805131 33436018

[37] Kramer AA Zimmerman JE. Assessing the calibration of mortality benchmarks in critical care: The hosmer-lemeshow test revisited. Crit Care Med (2007) 35(9):2052–6. doi: 10.1097/01.CCM.0000275267.64078.B0 17568333

[38] Minsky BD Pajak TF Ginsberg RJ Pisansky TM Martenson J Komaki R. INT 0123 (Radiation therapy oncology group 94-05) phase III trial of combined-modality therapy for esophageal cancer: high-dose versus standard-dose radiation therapy. J Clin Oncol: Off J Am Soc Clin Oncol (2002) 20(5):1167–74. doi: 10.1200/JCO.2002.20.5.1167 11870157

[39] Muijs CT Beukema JC Mul VE Plukker JT Sijtsema NM Langendijk JA. External beam radiotherapy combined with intraluminal brachytherapy in esophageal carcinoma. Radiothe Oncol J Eur Soc Ther Radiol Oncol (2012) 102(2):303–8. doi: 10.1016/j.radonc.2011.07.021 21885139

[40] Rishi A Zhang GG Yuan Z Sim AJ Song EY Moros EG. Pretreatment CT and (18) f-FDG PET-based radiomic model predicting pathological complete response and loco-regional control following neoadjuvant chemoradiation in oesophageal cancer. J Med Imaging Radiat Oncol (2021) 65(1):102–11. doi: 10.1111/1754-9485.13128 33258556

[41] Kao YS Hsu Y. A meta-analysis for using radiomics to predict complete pathological response in esophageal cancer patients receiving neoadjuvant chemoradiation. In Vivo (Athens Greece) (2021) 35(3):1857–63. doi: 10.21873/invivo.12448 PMC8193315 33910873

[42] Chen LL Nolan ME Silverstein MJ Mihm MC Sober AJ Tanabe KK. The impact of primary tumor size, lymph node status, and other prognostic factors on the risk of cancer death. Cancer (2009) 115(21):5071–83. doi: 10.1002/cncr.24565 19658184

[43] Leijenaar RTH Carvalho S Hoebers FJP Aerts HJWL van Elmpt WJC Huang SH. External validation of a prognostic CT-based radiomic signature in oropharyngeal squamous cell carcinoma. Acta Oncol (2015) 54(9):1423–9. doi: 10.3109/0284186X.2015.1061214 26264429

[44] Huang Y-Q Liang C-H He L Tian J Liang C-S Chen X. Development and validation of a radiomics nomogram for preoperative prediction of lymph node metastasis in colorectal cancer. J Clin Oncol Off J Am Soc Clin Oncol (2016) 34(18):2157–64. doi: 10.1200/JCO.2015.65.9128 27138577

[45] van Rossum PSN Fried DV Zhang L Hofstetter WL van Vulpen M Meijer GJ. The incremental value of subjective and quantitative assessment of 18F-FDG PET for the prediction of pathologic complete response to preoperative chemoradiotherapy in esophageal cancer. J Nucl Med (2016) 57(5):691–700. doi: 10.2967/jnumed.115.163766 26795288

[46] Jiang Y Chen C Xie J Wang W Zha X Lv W. Radiomics signature of computed tomography imaging for prediction of survival and chemotherapeutic benefits in gastric cancer. EBioMedicine (2018) 36:171–82. doi: 10.1016/j.ebiom.2018.09.007 PMC6197796 30224313

[47] Hong D Zhang L Xu K Wan X Guo Y. Prognostic value of pre-treatment CT radiomics and clinical factors for the overall survival of advanced (IIIB-IV) lung adenocarcinoma patients. Front Oncol (2021) 11:628982. doi: 10.3389/fonc.2021.628982 34123786 PMC8193844

[48] Kim JW Kim TH Kim JH Lee IJ. Predictors of post-treatment stenosis in cervical esophageal cancer undergoing high-dose radiotherapy. World J Gastroenterol (2018) 24(7):862–9. doi: 10.3748/wjg.v24.i7.862 PMC5807944 29467556

[49] Katada C Muto M Manabe T Boku N Ohtsu A Yoshida S. Esophageal stenosis after endoscopic mucosal resection of superficial esophageal lesions. Gastrointest Endosc (2003) 57(2):165–9. doi: 10.1067/mge.2003.73 12556777

[50] Straub JM New J Hamilton CD Lominska C Shnayder Y Thomas SM. Radiation-induced fibrosis: mechanisms and implications for therapy. J Cancer Res Clin Oncol (2015) 141(11):1985–94. doi: 10.1007/s00432-015-1974-6 PMC4573901 25910988

[51] Ahlberg A al-Abany M Alevronta E Friesland S Hellborg H Mavroidis P. Esophageal stricture after radiotherapy in patients with head and neck cancer: experience of a single institution over 2 treatment periods. Head Neck (2010) 32(4):452–61. doi: 10.1002/hed.21201 19672963

[52] Abu-Ghanem S Sung CK Junlapan A Kearney A DiRenzo E Dewan K. Endoscopic management of postradiation dysphagia in head and neck cancer patients: A systematic review. Ann Otol Rhinol Laryng (2019) 128(8):767–73. doi: 10.1177/0003489419837565 30895823

[53] Agarwalla A Small AJ Mendelson AH Scott FI Kochman ML. Risk of recurrent or refractory strictures and outcome of endoscopic dilation for radiation-induced esophageal strictures. Surg Endosc (2015) 29(7):1903–12. doi: 10.1007/s00464-014-3883-1 PMC4385000 25277484

[54] Ng TM Spencer GM Sargeant IR Thorpe SM Bown SG. Management of strictures after radiotherapy for esophageal cancer. Gastrointest Endosc (1996) 43(6):584–90. doi: 10.1016/s0016-5107(96)70196-7 8781938

[55] Xie CY Pang CL Chan B Wong EY Dou Q Vardhanabhuti V. Machine learning and radiomics applications in esophageal cancers using non-invasive imaging methods-a critical review of literature. Cancers (2021) 13(10). doi: 10.3390/cancers13102469 PMC8158761 34069367

[56] Li M Zhang X Zhao F Luo Y Kong L Yu J. Involved-field radiotherapy for esophageal squamous cell carcinoma: theory and practice. Radiat Oncol (London England) (2016) 11:18. doi: 10.1186/s13014-016-0589-7 PMC4743321 26846932

[57] Fan C-Y Su Y-F Huang W-Y Chao H-L Lin K-T Lin C-S. Definitive radiotherapy dose escalation with chemotherapy for treating non-metastatic oesophageal cancer. Sci Rep (2018) 8(1):12877. doi: 10.1038/s41598-018-31302-y 30150679 PMC6110762

[58] Yu W Cai X-W Liu Q Zhu Z-F Feng W Zhang Q. Safety of dose escalation by simultaneous integrated boosting radiation dose within the primary tumor guided by (18)FDG-PET/CT for esophageal cancer. Radiotherapy Oncol J Eur Soc Ther Radiol Oncol (2015) 114(2):195–200. doi: 10.1016/j.radonc.2014.12.007 25586952

[59] Abbasi M El Hanandeh A. Forecasting municipal solid waste generation using artificial intelligence modelling approaches. Waste Manage (2016) 56:13–22. doi: 10.1016/j.wasman.2016.05.018 27297046

[60] Nguyen XC Nguyen TTH La DD Kumar G Rene ER Nguyen DD. Development of machine learning - based models to forecast solid waste generation in residential areas: a case study from Vietnam. Resour Conserv Recycling (2021) 167:105381. doi: 10.1016/j.resconrec.2020.105381

