# Supplementary figures and images for: A combined predicting model for benign esophageal stenosis after simultaneous integrated boost in esophageal squamous cell carcinoma patients (GASTO1072)
Source: Front Oncol. 2022 Dec 22;12:1026305. doi: 10.3389/fonc.2022.1026305 (PMC10107369; doi:10.3389/fonc.2022.1026305)

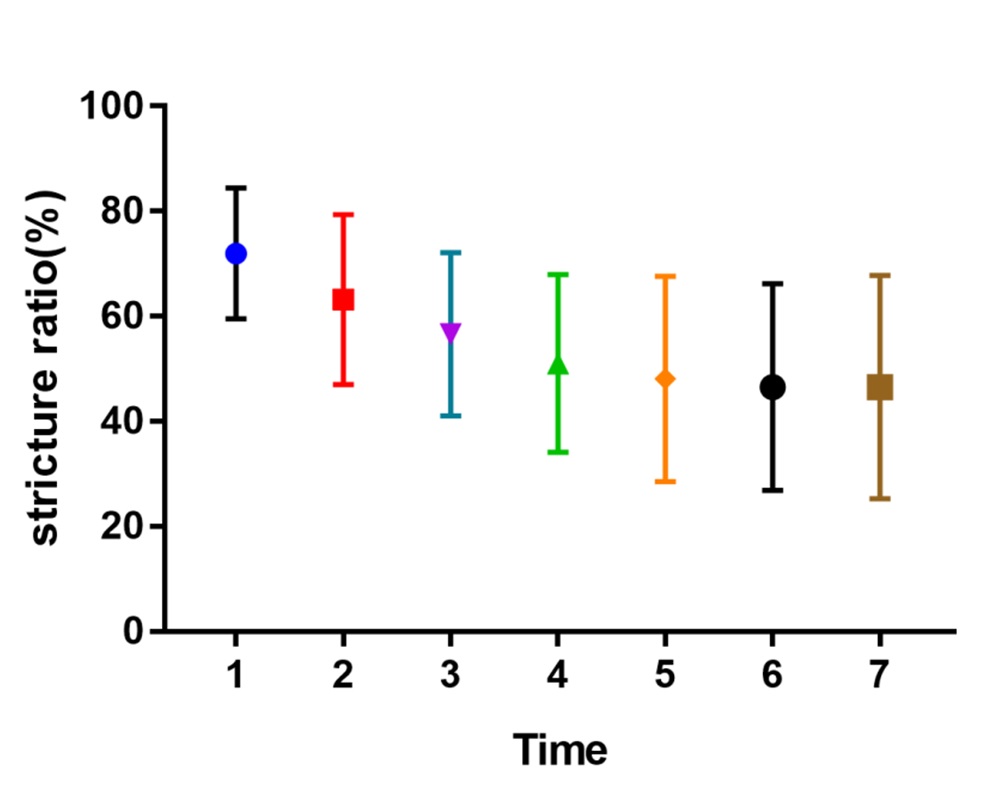

Supplement: Supplementary Figure 1 — The change in the mean esophageal stenotic ratio of 65 patients before treatment to 1 year after treatment. Seven points in x axis represent before treatment, twentieth fraction, complete treatment, 3, 6, 9, and 12 months after completing treatment. [file Image_1.jpeg]

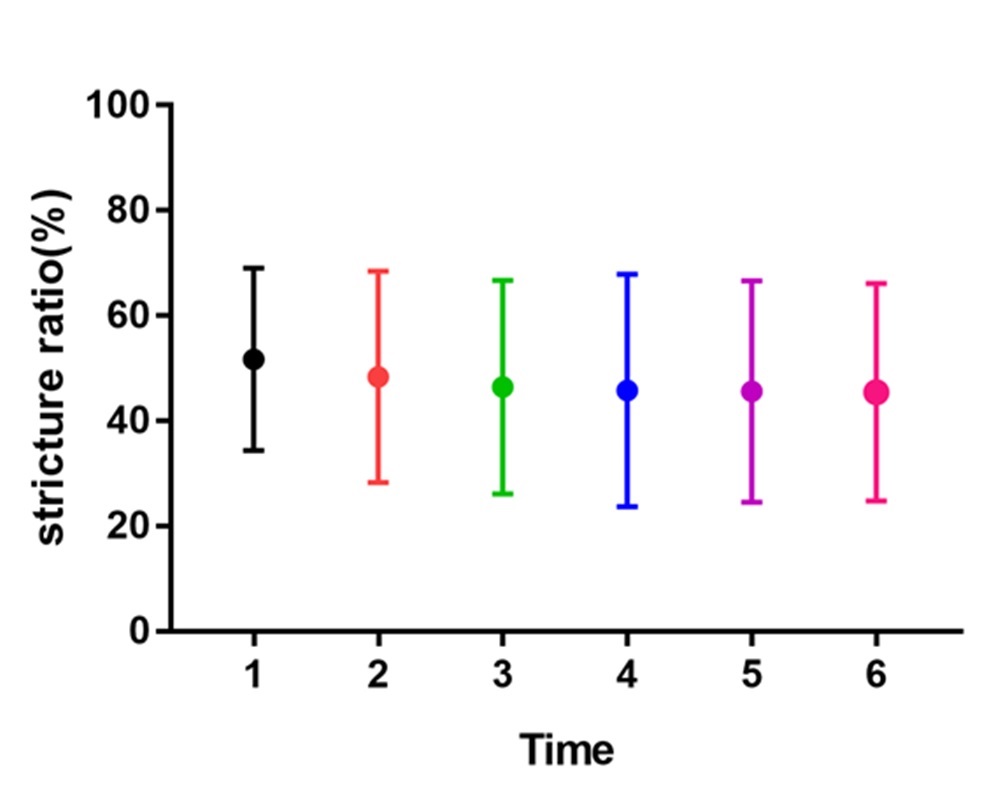

Supplement: Supplementary Figure 2 — The change in the mean esophageal stenotic ratio of 48 patients from 3 to 18 months after treatment. Six points in x axis represent 3, 6, 9, 12, 15, and 18 months after completing treatment. [file Image_2.jpeg]
