# Supplementary material for: A combined predicting model for benign esophageal stenosis after simultaneous integrated boost in esophageal squamous cell carcinoma patients (GASTO1072)
Source: Front Oncol. 2022 Dec 22;12:1026305. doi: 10.3389/fonc.2022.1026305 (PMC10107369; doi:10.3389/fonc.2022.1026305)
Supplement: Supplementary file 3 [file Table_1.docx]

**Supplementary Materials**

**Supplementary Tables**

**Table S1. The change of the esophageal stenotic ratio in 65 EC patients from before treatment to 1 year after treatment**

| Time | Stenotic ratio（%） | Mean±SD |
| --- | --- | --- |
| Pre-treatment | 41.80-95.30 | 72.00±12.48 |
| 20th fraction of radiotherapy | 27.00-94.00 | 63.22±16.16 |
| End of treatment | 16.30-82.40 | 56.59±15.56 |
| 3 months after treatment | 13.40-85.10 | 51.02±16.92 |
| 6 months after treatment | 12.10-90.40 | 48.11±19.55 |
| 9 months after treatment | 13.50-92.60 | 46.56±19.68 |
| 12 months after treatment | 5.20-90.10 | 46.53±21.22 |

Abbreviations: EC=esophageal cancer, SD=Standard deviation

**Table S2. The change of the esophageal stenotic ratio in 48 EC patients in 18 months after treatment**

| Time | Stenotic ratio（%） | Mean±SD |
| --- | --- | --- |
| 3 months after treatment | 20.00-85.10 | 51.71±17.30 |
| 6 months after treatment | 15.30-88.60 | 48.36±20.06 |
| 9 months after treatment | 14.20-92.60 | 46.42±20.29 |
| 12 months after treatment | 5.20-90.10 | 45.81±22.08 |
| 15 months after treatment | 7.40-91.30 | 45.59±21.07 |
| 18 months after treatment | 7.60-90.20 | 45.45±20.70 |

Abbreviations: EC=esophageal cancer, SD=Standard deviation

**Table S3. The collinearity diagnosis for the 11 features.**

| Variables | Collinearity statistics | |
| --- | --- | --- |
|  | Tolerance | VIF |
| ECI | .618 | 1.617 |
| LLT | .145 | 6.891 |
| NEWT>1cm | .172 | 5.805 |
| max | .500 | 2.001 |
| SphericalDisproportion | .370 | 2.700 |
| IdistCent | .628 | 1.593 |
| Informaiton Measure of Correlation2_GLCM | .499 | 2.005 |
| Run_Percentage _GLRLM | .116 | 8.640 |
| Texture_Strength_NGTDM | .367 | 2.723 |
| Small_Zone_Emphasis_GLSZM | .505 | 1.982 |

VIF, variance inflation factor; GLCM, grey level co-occurrence matrices; NGTDM, neighborhood grey-tone difference matrices; GLSZM, grey level size-zone matrices.
